# Partial agenesis of the dorsal pancreas with features of chronic pancreatitis: A case report

**DOI:** 10.1016/j.radcr.2024.10.050

**Published:** 2024-11-10

**Authors:** Naqibullah Foladi, Farhad Farzam, Sayed Mohammad Milad Fekrat, Najibullah Rahil, Mohammad Javid Karimy

**Affiliations:** a Radiology Department, Wyagal Radiology Center, Kabul, Afghanistan; b Radiology Department, Amir Hajizada Hospital, Kabul, Afghanistan; c Kabul University of Medical Sciences, Kabul, Afghanistan; d Maiwand Hospital, Kabul, Afghanistan; e Wyagal Radiology Center, Kabul, Afghanistan

**Keywords:** Case report, Partial agenesis of the dorsal pancreas, Dorsal pancreatic agenesis

## Abstract

Complete agenesis of the pancreas or the absence of its ventral portion is incompatible with life. However, agenesis of the dorsal pancreas is a relatively benign condition. Partial agenesis of the dorsal pancreas (ADP) arises from abnormal embryogenesis, although the exact etiology remains unknown. In cases of complete dorsal pancreas agenesis, the head, body, tail, minor duodenal papilla, and duct of Santorini are absent, while partial agenesis involves only the minor duodenal papilla and Santorini duct. We present the case of a 60-year-old woman with nonspecific abdominal pain, referred for an abdominal CT scan. The scan revealed the uncinate process and partial head of the pancreas, including the ducts of Wirsung and Santorini, while the remainder of the dorsal pancreas was absent. These findings are consistent with partial ADP. Additionally, multifocal dense calcifications were noted in the ventral pancreas, suggesting chronic pancreatitis. No associated anomalies or additional symptoms were detected. Partial ADP is a scarce condition, and the coexistence of chronic pancreatitis further contributes to its uniqueness. The etiology of chronic pancreatitis in this case remains unclear. The increasing recognition of ADP in recent years is likely due to advances in radiological imaging. Imaging plays a crucial role not only in diagnosing ADP but also in assessing prognosis and detecting any associated anomalies. While specific treatment is unnecessary in the absence of other anomalies, annual screening is recommended due to the potential risk of malignancy in the ventral pancreas.

## Introduction

There are various pancreatic anomalies, but partial dorsal pancreatic agenesis is an extremely uncommon anomaly, that arises from abnormal embryogenesis [[1], [2], [3], [4], [5]]. However, complete absence or agenesis of the ventral pancreas is not compatible with life [1,2,5]. Dorsal pancreatic agenesis can be full or partial. The duct of Santorini and minor duodenal papilla and part of the body of the pancreas are present in partial agenesis of the pancreas. In contrast, complete agenesis of the dorsal pancreas represents an absence of minor duodenal papilla, duct of Santorini, the body and tail of the pancreas [[4], [5], [6]]. Patients with dorsal pancreatic agenesis are mostly asymptomatic, however, a few of the cases can present with abdominal pain [6], diabetes, or chronic pancreatitis [2]. The discovery of the first case of dorsal pancreatic agenesis took place in 1911 during a postmortem examination [[1], [2], [3], [4], [5], [6]]. Since then about 100 cases have been reported in the English literature [2,5]. The authors represent a 60-year-old woman complaining of nonspecific pain in the abdomen, for which an abdomen contrast-enhanced CT scan revealed partial agenesis of the dorsal pancreas with features of chronic pancreatitis.

## Case presentation

A 60-year-old female experiencing chronic nonspecific abdominal discomfort was referred for an abdomen contrast-enhanced CT scan by the Department of Radiology.

There were no significant abnormal findings during a physical examination. There were no noteworthy findings in the patient's medical, surgical, familial, and psychosocial history.

**Fig. 1:**
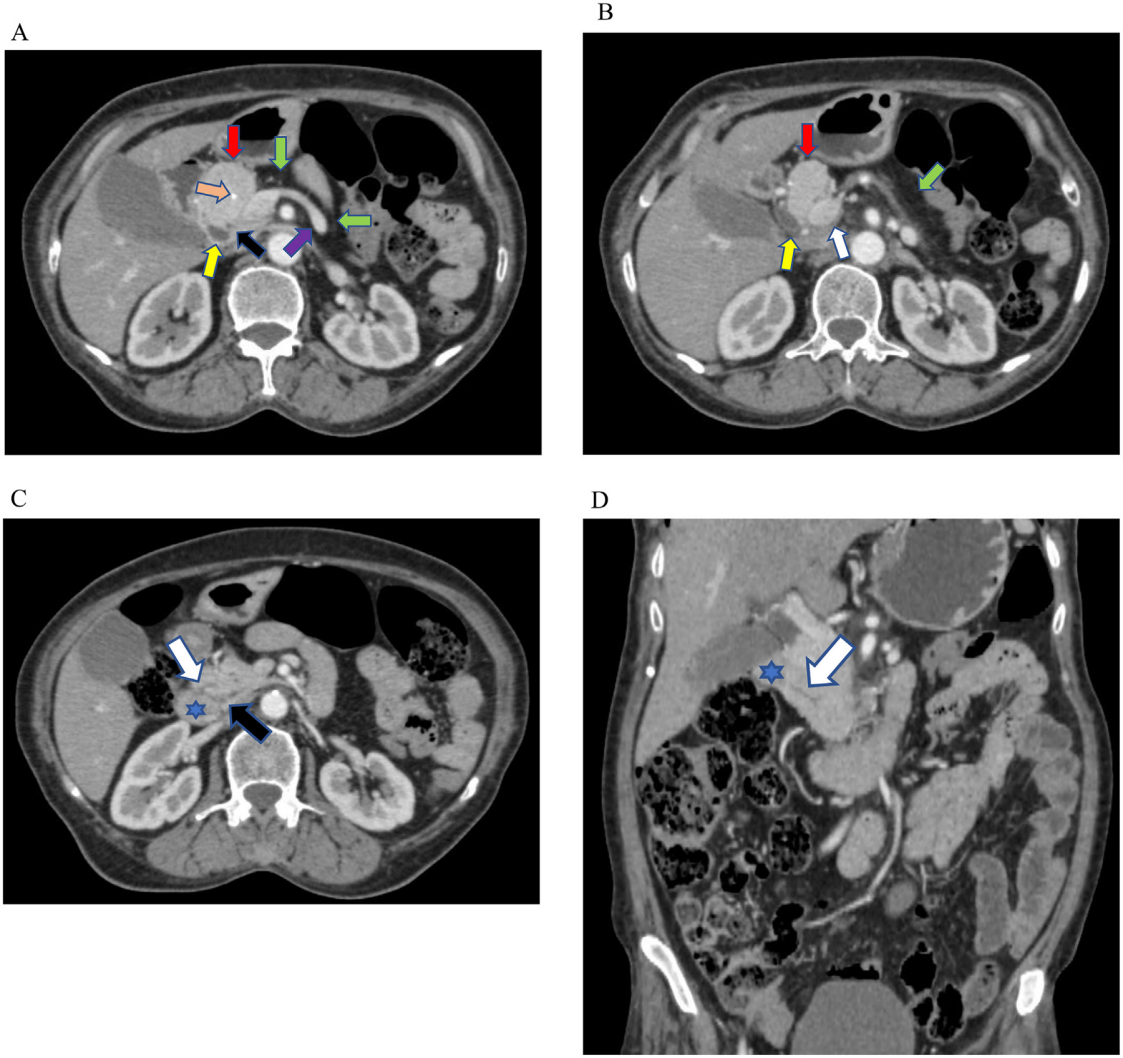
(A-D) Contrast-enhanced selected axial and coronal CT images of the abdomen revealing the uncinate process and part of the head of the pancreas. (red arrow). The rest of the pancreas appears absent, instead occupied by the retroperitoneal fat (green arrows) anterior to the splenic vein (purple arrow). The duct of Santorini accessing the minor papilla (white arrow) and the duct of Wirsung (black arrow) communicating with the major duodenal papilla (blue arrow) of the duodenum (blue asterisk). Foci of dense calcifications present within the ventral pancreas (orange arrow). The distal common bile duct (yellow arrow) and portal vein (white arrow) are noted.

**Fig. 2:**
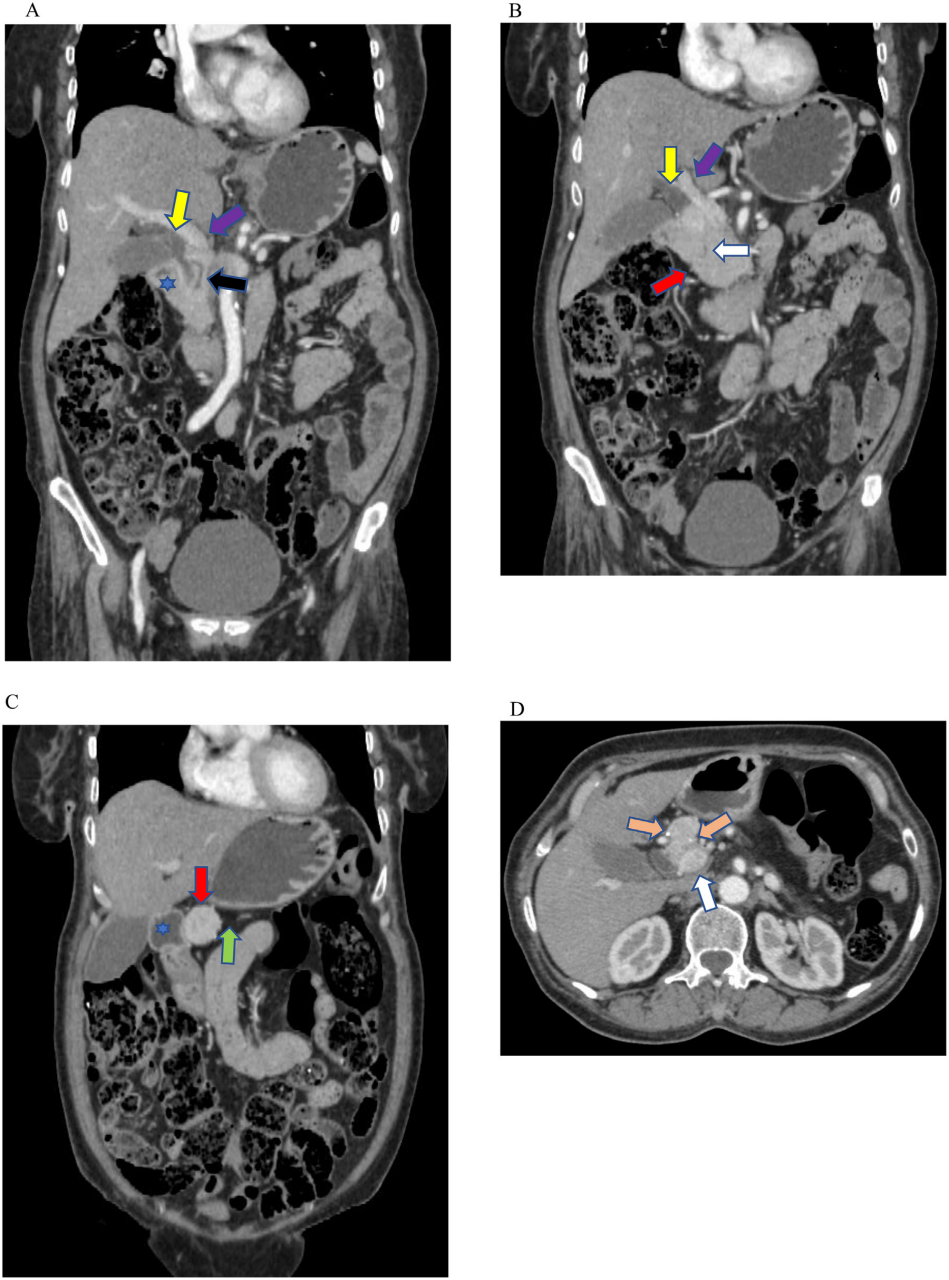

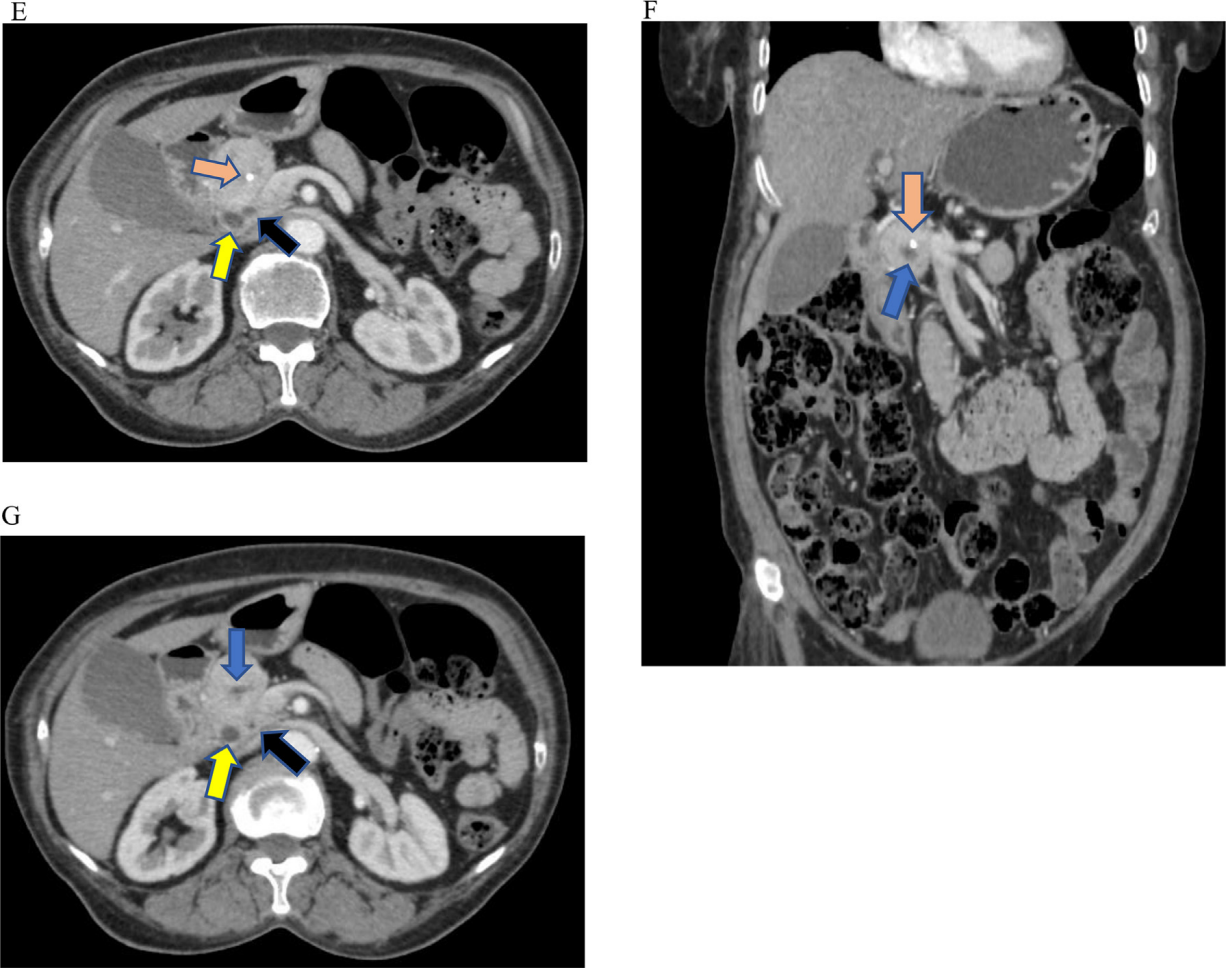
(A-G) Contrast-enhanced selected axial and coronal CT images of the abdomen demonstrating variable-sized calcifications at the uncinate/head of the pancreas (orange arrows) and an irregular cavity near the calcification (blue arrows). A portion of the ventral pancreas (red arrow) is appreciated. The retroperitoneal fat (green arrow) occupies the anatomic location of the dorsal pancreas. The Wirsung (black arrow) and Santorini ducts (white arrow) can be visualized clearly. Anatomic structures including the common bile duct (yellow arrow), duodenum (blue asterisk), and portal vein (purple arrow) are visible.

Abdomen CT demonstrated the uncinate process and part of the head of the pancreas. However, the remaining dorsal portion of the pancreas was absent. Instead, the retroperitoneal fat replaces the lacking portion of the pancreas (Figs. 1A-D). The ducts of Santorini and Wirsung were visible communicating with minor and major duodenal papillae, respectively. There were multifocal dense calcifications with an irregular cavity in the ventral pancreas, marking the features of chronic pancreatitis (Figs. 2A-G). No associated abnormal findings in the abdomen were found such as heterotaxy, polysplenia, and renal cysts. There was no mass in the pancreas or features of pancreatic lipomatosis as the ducts could not be visualized in the agenesis of the dorsal pancreas (ADP). The imaging features were characteristic of partial agenesis of the dorsal pancreas (ADP) with features of chronic pancreatitis. The pancreatic enzymes were in the normal range. The patient didn't have any signs or symptoms of diabetes. The HbA1c and random blood glucose levels were within normal range. The Department of Radiology couldn't access the patient for the follow-up.

## Discussion

Complete or partial agenesis of the dorsal pancreas (ADP) is an exceptional congenital abnormality associated with irregular embryogenesis where the dorsal pancreatic bud fails to develop into the body and tail of the pancreas [[1], [2], [3], [4], [5], [6]]. During the early stages of gestation, around the seventh week, the ventral bud rotates backward and fuses with the dorsal bud, a process critical for forming the pancreas [1,2,4]. The ventral bud forms the head and the uncinate process is drained by the Wirsung duct into the major duodenal papilla. The dorsal bud forms the part of the head, the body, and the tail of the pancreas, which drains through the duct of Santorini into the minor duodenal papilla [[2], [3], [4], [5], [6]]. Disruptions in the normal embryonic process can result in partial or complete absence of the dorsal pancreas. In complete agenesis, the pancreatic body, tail, and duct of Santorini and minor papilla are absent. In partial agenesis, the minor papilla and accessory remain [[1], [2], [3], [4], [5], [6]].

The first recorded case of ADP was in 1911 during a postmortem examination [[4], [5], [6]]. Since then about 100 cases have been documented in the scientific literature [1,2,5]. ADP can occur sporadically or follow autosomal dominant or X-linked dominant inheritance [2,7]. However, in our case, there was not any associated family history. The precise cause and mechanism behind the dorsal pancreatic agenesis remain unidentified. Lately, the changes in certain signaling pathways, namely hedgehog [2,4], and retinoic acid pathways, have been recognized as potential contributors. Notably, the aforementioned signaling pathways have also been considered to play a role in the pathogenesis of the ductal adenocarcinoma of the pancreas [2,6,7].

Typically, most patients with dorsal pancreatic agenesis exhibit no noticeable symptoms [[1], [2], [3], [4], [5], [6]]. However, if symptoms occur, epigastric pain stands as the primary complaint [[1], [2], [3], [4]]. The epigastric pain is more localized and worsens after meals [2]. In our case, the patient had chronic nonspecific abdominal pain. It is worth noting that nearly half of ADP patients may experience hyperglycemia, which can be linked to underlying diabetes mellitus [1,[3], [4], [5], [6]]. Predominantly, the islet cells are located in the dorsal pancreas. Thus, their lack causes a disorder in glucose metabolism [3,5]. In our case the blood glucose level was normal and there wasn't any history of diabetes. Patients with ADP are also at risk of developing acute or chronic pancreatitis due to risk factors such as dysfunction of the Oddi sphincter, oversecretion of enzymes, and elevated pressures in the duct of the pancreas. In certain cases, chronic pancreatitis can lead to the development of pseudocysts [1,6]. There is also the possibility of a tumor development in the agenesis of the dorsal pancreas such as intraductal papillary mucinous neoplasm (IPMN), or cystic lesions. These tumors are thought to arise as a consequence of long-term chronic pancreatitis [5]. In our case, the calcifications observed in the ventral pancreas were indicative of chronic pancreatitis.

ADP can be associated with various other developmental abnormalities, including polysplenia syndrome, heterotaxy, splenic ectopia [6], malrotation of the bowel, coarctation of the aorta, tetralogy of Fallot, and atrioventricular valvular abnormalities [1,2]. In our case, no such anomalies were detected.

The differentials that can mimic and share similar features to ADP are pancreatic carcinoma, autodigestion due to chronic pancreatitis, pancreatic divisum, lipomatosis, and pseudoagenesis. Pseudoagenesis is best explained by replacing pancreatic tissue with fat following necrohemorrhagic pancreatitis [1]. In our case, there was no pancreatic mass, lipomatosis, or any association of anomalies.

There are multiple modalities to diagnose ADP, such as ultrasound, CT scan MRI [1], and ERCP [6] Ultrasound may show echolucent pancreatic head but it may be insufficient in adults due to overlying bowel gas or body habitus. CT and MRI, particularly MRCP, provide more detailed imaging revealing the absence of the dorsal pancreas and aid in diagnosis. On a CT scan, the characteristic signs include the “dependent stomach” or the “dependent intestine” signs, where the stomach or the small bowel occupies the space where the pancreas should be located [1]. MRI, including MRCP, can be appreciated as it delineates the pancreatic ductal system and demonstrates an absent dorsal duct in patients with ADP. Unless there is suspicion of pancreatic malignancy, the treatment is typically symptomatic [1,4]. In our case, both ducts of Wirsung and Santorini were clearly visible communicating with major and minor duodenal papillae in the CECT examination, confirming the diagnosis of the partial agenesis of the dorsal pancreas.

Partial agenesis of the dorsal pancreas with chronic pancreatitis of the ventral pancreas is an exceptional combined abnormality. The rise in the cases of ADP is believed to be due to the development of imaging modalities in the past decades. Although agenesis of the dorsal pancreas is a benign condition, the importance of imaging modalities in detecting pancreatic anomalies and associated abnormalities is highly appreciated. Agenesis of the dorsal pancreas doesn't require any treatment, but annual screening may be recommended due to the potential for malignancy in the ventral pancreas.

## Ethics approval and consent to participate

The manuscript has an ethical review exemption from the Ethical Review Committee (ERC) of the authors’ institution (Amir Hajizada Hospital - {AHH}) as case reports are exempted from review according to the institutional ethical review committee's policy. Written consent is obtained from the participants for publishing the case.

## Availability of data and materials

Data sharing is not applicable to this article as no datasets were generated or analyzed during the current study (as this is a case report).

## Author contributions

All authors have participated sufficiently in the submission and take public responsibility for its content. NF: writing and editing the manuscript and selecting images, as well as corresponding with the journal. FF: selecting the case and revising the manuscript. SMMF: writing and editing the manuscript. MJK: revising and editing the manuscript. All of the authors have read and approved the final manuscript.

## Patient consent

Written informed consent was obtained from the patient for publication of this case report and any accompanying images. A copy of the written consent is available for review by the Editor of this journal.
